# The Livingstone Cottage Hospital, Dartford

**Published:** 1895-03-09

**Authors:** 


### EDITOR'S LETTER-BOX.

[Our correspondents are reminded that proxility is a great bar to publication, and that brevity of style and conciseness of statement greatly facilitate early insertion.]

### THE LIVINGSTONE COTTAGE HOSPITAL, DARTFORD.

MR. G. H. TAIT writes: In your notice of the Livingstone Cottage Hospital in your issue of 16th ult. there are one or two criticisms which I think would not have been made had the writer seen the buildings or else the working drawings, and I would ask you kindly to note the following explanations. (1) The room suggested as a consulting-room is intended for interviewing the nurse or doctor as well as for dispensing, but at present the arrangements for dispensary work have not been completed. (2) The surgery is also the operation-room, and has good top light in addition to windows. (3) The wards are well lighted from the ends (east and west), and for ventilation, in addition to side windows shown on plan, there are large windows over the flat roofs of the lobbies marked A.A. In the corners of wards marked are large perforated zinc openings near ceiling level communicating with a skylight fixed in roof above the ceiling of the duty-room which effectually prevents any stagnation of air in that corner. (4) The duty-room had only "spy' windows commanding the south rooms, but it was deemed advisable to put small doors instead in case of a special patient requiring to be treated there, and so afford easy access. These special wards were intended for convalescent rooms, and should have been named so, and for that reason were purposely placed on south side with a beautiful view for miles up the Darenth Valley. The only reason for showing these rooms as "special" wards was to denote the extent to which we could accommodate patients on a pinch, or under other necessary circumstances. (5) It is hoped that the wards will not be filled with their full complement for a long time, but even if two nurses are required at the same time in the duty-room it is amply large for them. Perhaps these few notes will explain the points referred to satisfactorily.

*** Mr. Tait's explanations do not convince us that the lighting and ventilation of his wards are all that can be desired. We cannot regard a window over a lead flat, and whose sill must be at least eight feet above the floor, as a satisfactory way of lighting a ward; neither do we think a perforated zinc opening, communicating with a skylight over the duty-room, a good mode of ventilation. The intentions of the committee or their architect seem to have got a little mixed on the question of the ultimate use of the "special wards" or "convalescent room." In a small hospital of this kind two convalescent rooms are unnecessary, and if used as special wards the nursing accommodation is, as pointed out, insufficient, and the insufficiency depends, not, as Mr. Tait seems to think, on the size of the duty-room, but upon the fact that there is only sleeping accommodation for two nurses.—ED. *T. H.*

